# A Survey of Dummy-Based Location Privacy Protection Techniques for Location-Based Services

**DOI:** 10.3390/s22166141

**Published:** 2022-08-17

**Authors:** Shiwen Zhang, Mengling Li, Wei Liang, Voundi Koe Arthur Sandor, Xiong Li

**Affiliations:** 1 School of Computer Science and Engineering, Hunan University of Science and Technology, Xiangtan 411201, China; 2 School of Computer Science and Cyber Engineering, Guangzhou University, Guangzhou 510006, China; 3 School of Computer Science and Engineering, University of Electronic Science and Technology of China, Chengdu 611731, China

**Keywords:** location privacy, privacy protection, dummy location

## Abstract

As smart devices and mobile positioning technologies improve, location-based services (LBS) have grown in popularity. The LBS environment provides considerable convenience to users, but it also poses a significant threat to their privacy. A large number of research works have emerged to protect users’ privacy. Dummy-based location privacy protection solutions have been widely adopted for their simplicity and enhanced privacy protection results, but there are few reviews on dummy-based location privacy protection. Or, for existing works, some focus on aspects of cryptography, anonymity, or other comprehensive reviews that do not provide enough reviews on dummy-based privacy protection. In this paper, the authors provide a review of dummy-based location privacy protection techniques for location-based services. More specifically, the connection between the level of privacy protection, the quality of service, and the system overhead is summarized. The difference and connection between various location privacy protection techniques are also described. The dummy-based attack models are presented. Then, the algorithms for dummy location selection are analyzed and evaluated. Finally, we thoroughly evaluate different dummy location selection methods and arrive at a highly useful evaluation result. This result is valuable both to users and researchers who are studying this field.

## 1. Introduction

In the United States, a large majority (90%) of smartphone owners used location-based services [1]. Locations are being used more frequently than ever before since the global pandemic. For example, the government should keep a record of every location ever visited, and track the whereabouts of people who have tested positive for COVID-19 to determine where the virus is likely to spread next [2]. Furthermore, location-based services will continue to gain attention and become more widely used in the future. According to Federica Laricchia, the annual worldwide blue-tooth location service device shipments reached 183 million units in 2021, with yearly shipments expected to reach 568 million units in 2026 [3].

**Figure 1:**
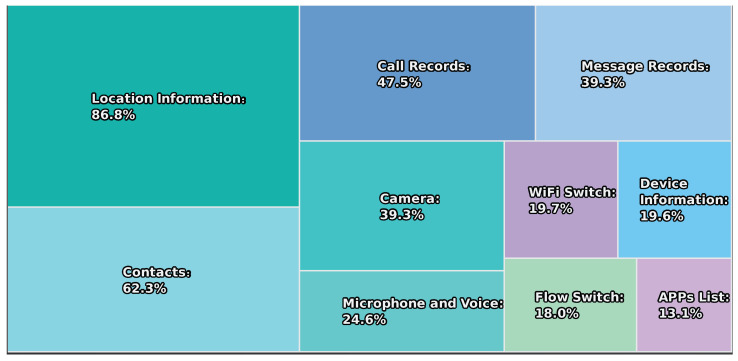
Permissions to install and use mobile apps.

While location-based services are widely used and provide significant convenience to users and society, they also pose a significant threat to privacy. According to risk-based security [4], the total amount of global data leakage in 2021 has reached 22 billion, which is about 14.5 billion less than in 2020. However, such an amount also quantifies the second highest year for confidential data leakage since 2005. As shown in Figure 1, a survey conducted by the China Consumers Association [5] in 2018 found that more than 80% of respondents had experienced personal information leakage. Moreover, it is common for mobile apps to collect excessive amounts of personal information, while location data have evolved into a type of profitable resource.

In most cases, location data are linked to other sensitive attributes, such as health status, home address, behavioral habits, and other privacy concerns. As a result, protecting the location information of smartphone users, specially those who use location-based services, is critical and urgent.

Dummy refers to the method of adding multiple dummy locations and sending them to the LBS server along with the real query location to blur the real location. Domestic and foreign researchers have proposed a variety of location privacy protection schemes based on dummy. For example, Kido et al. proposed the first dummy-based location privacy protection techniques in the literature [6]. They generated dummy locations at random using the random walk model. Hara et al. [7] designed a method for selecting dummies that takes real-world constraints into account, such as excluding places where people are unlikely to exist. Niu B. [8] proposed a Cir-dummy- and Grid-dummy-based dummy location selection algorithm. Shu C. [9] proposed two new dummy selection algorithms, MaxMinDistDS and Simp-MaxMinDistDS, that take both the location semantic diversity and the physical dispersion into account.

There are numerous dummy-based schemes being made to deal with location privacy. However, reviews for dummy-based schemes are relatively rare, and some focus on aspects relating to cryptography [10], anonymity [11], or other comprehensive reviews [12], which focus on the whole picture, but there are not enough review on dummy-based privacy protection. In addition, these reviews fail to clarify the relationship between the level of privacy (LoP), the quality of service (QoS), and system overhead. These are struggle to explain the difference and relationship between dummy and other location privacy protection techniques, as well as analyze and summarize the dummy location attack model and how to choose dummies. Therefore, such studies cannot help readers understand the up-to-date challenges of dummy-based privacy protection brought on by attackers’ expanding background knowledge and the intersection between LBS and other emerging technologies.

In this paper, we make a review of dummy-based location privacy protection techniques for location-based services. The main contributions are as follows.

- First, we distinguish the relationship between the LoP, the QoS, and the system overhead. Additionally, we make an overall comparison of several representative methods of location privacy protection techniques. Then, we describe the merits of dummy-based location privacy protection on LBS. Meanwhile, a summary of the major attacks on dummy-based location privacy protection techniques is also included.
- Second, we systematically and comprehensively analyze and summarize the ways of selecting dummies on three aspects, namely the query probability, the physical dispersion, and the semantic diversity of locations.
- Third, we provide an overview of the methods for achieving query probability, physical dispersion, and semantic diversity while choosing dummies. Furthermore, we make comparative analysis to indicate the different privacy protection advantages of different selection rules when choosing dummies. Results of this comparative analysis can be of benefit both to users and researchers who are studying this field.

The remainder of this paper is organized as follows. Section 2 gives an overview of location privacy protection, and Section 3 provides a summary of the attack model of dummy-based location privacy protection techniques. Section 4 describes the system architecture and privacy protection methods, and also gives a detailed analysis and summary of how to choose a dummy location. Finally, in Section 5, we conclude our work.

## 2. Overview of Location Privacy Protection

In this section, we first introduce the key issues in location privacy protection and location privacy protection techniques. Then, we describe the difference and connection of various location privacy protection techniques, and finally make a comparison between them.

### 2.1. Location Privacy Protection

When users use LBS, their location privacy is compromised to some degree due to dishonest or semi-trusted LBS servers serving private interests. Nonetheless, because location privacy is closely related to explicit sensitive information, other implicit sensitive information about users is also leaked. Take, for example, John. He has been feeling uneasy lately, so he decides to go to the hospital to find out what’s wrong with his body. However, because he does not want others to know about his medical condition, the hospital’s location is important to him. In reality, “where are you staying” reveals the privacy of “what are you doing”. Similarly, the user’s historical location data expose the locations he frequently shows up at, and the routes he travels a lot by, which leads to his home address, behavioral habits, work nature, and other sensitive information he cares about potentially being leaked [13]. Therefore, there is no doubt that it is definitely vital to protect a user’s location privacy.

### 2.2. Key Issues of Location Privacy Protection

When it comes to location privacy protection, it is naturally necessary to consider the connection between LoP, QoS, and the system overhead [14].

#### 2.2.1. Issue on the Relationship between LoP and QoS

In location privacy protection, high LoP and QoS cannot be satisfied at the same time. To obtain location services, users must submit their location to the service provider in some way, which risks exposing their private information. Many techniques, such as using cloaking areas instead of the real location, adding noise to the real location, and so on, sacrifice some degree of location accuracy for higher LoP. However, if the location accuracy is too low to meet users’ demands, availability will suffer, and privacy protection will be rendered ineffective. Furthermore, the requirements for location service quality vary depending on the user. Users who request to query a specific point of interest will be more concerned with QoS. Users seeking hospital location service, on the other hand, will be more concerned with their location information. As a result, they are willing to sacrifice some service quality in exchange for a higher LoP. Therefore, understanding the relationship between QoS and LoP is one of the most crucial matters in location privacy protection.

#### 2.2.2. Issue on the Relationship between QoS and System Overhead

With the advent of the “fast” era, people are more concerned with speed, even when it comes to location privacy protection. People desire faster response times and lower latency. When a user initiates a query request, the user experience will suffer if the response time is too slow. However, the majority of existing studies improve LoP without taking system overhead into account, or at the expense of a significant increase in system overhead to achieve a minor improvement in LoP. Simultaneously, the costs of communication, storage, and computation, as well as the loss of precision, all have an impact on the user experience due to the limited resources on the user’s device. For example, a large amount of computation cost slows down the processing speed of mobile devices, a large amount of communication cost raises the extra cost for users, and a large amount of electricity overhead affects outdoor use of mobile devices, ultimately hindering the development of location service [15]. Understanding the relationship between LoP and the system overhead is therefore another critical issue in location privacy protection.

### 2.3. Location Privacy Protection Techniques

Researchers proposed numerous approaches to protect location privacy, such as [16,17,18,19]. In general, location privacy protection techniques can be divided into four categories [20]: obfuscation [21], encryption [22,23,24], cache and collaboration [25], and anonymity mechanisms [26].

#### 2.3.1. Location Privacy Protection Techniques Based on Obfuscation

Location privacy protection techniques based on obfuscation refer to the necessary disruption to the original location information in an LBS query in order to prevent the attacker from obtaining the user’s true location while also ensuring that the user can acquire unrestricted services. Dummy [6], spatial cloaking [27,28], differential privacy [29], and other obfuscation techniques can reduce the accuracy of location information. The dummy method adds multiple dummies and sends them to the LBS server along with the real query location to blur the real location. To protect users’ location privacy, Li et al. [30] proposed an attribute-aware privacy protection scheme (APS). The Voronoi dividing algorithm (VDA) and the dummy determining algorithm (DDA) are two algorithms included in APS. The VDA algorithm divides the local map into different Voronoi polygons to ensure that the selected dummy locations are scattered, whereas the DDA algorithm chooses dummy locations based on the four-color mapping theorem to ensure that dummy locations differ in attributes. The classical dummy method, which was later extended to trajectory, is frequently used to solve the single location problem. Ni et al. [31] proposed an R-constrained dummy trajectory-based privacy-preserving algorithm (RcDT). The generated dummy locations are in a specific range close to the real location because the generating range R of the dummy location is constrained. Furthermore, by constraining the exposure risk of each dummy location and trajectory, dummy trajectories with a higher similarity to the real trajectory are generated. Differential privacy protects location privacy by adding an appropriate number of noises to the returned value of the query function [29]. Several recent studies [32,33] have investigated the use of differential privacy in location protection. The concept of protecting user locations within a radius R, whose privacy level is dependent on R, is formally defined by the term of geographical indiscernibility [32]. To increase the user’s LoP, controlled random noise is added to their location. In general, using the obfuscation strategy will result in a significant loss of precision in query results.

#### 2.3.2. Location Privacy Protection Techniques Based on Encryption

To achieve the privacy goals, the cryptographic approach adopts encryption technology to make the user’s query content and location information completely transparent to the LBS server. While ensuring QoS, this technique does not reveal any user’s location information, ensuring stricter privacy protection. Private information retrieval (PIR) [34,35] is a popular encryption method. PIR prevents the server (the database owner) from determining the user’s point of interest and drawing additional conclusions about the client’s private information by ensuring that the server (the database owner) cannot determine the correct query object when the user requests the database. Paulet et al. [34] obtained and decrypted location data using a PIR-based protocol. The user’s location information was kept private because the server was unable to determine it. The PIR method ensures the confidentiality of the entire communication process (user request, information retrieval, and result return process). However, the issue of over-collected storage and computation overhead in PIR needs to be investigated further. The primary challenge in using PIR is developing a good retrieval strategy and index structure. However, because the LBS server must store the entire map information of the local map, the server’s limited storage space as well as retrieval efficiency make PIR only applicable to a small space range at the present time.

#### 2.3.3. Location Privacy Protection Techniques Based on Collaboration and Cache

Collaboration and caching cut down the time spent communicating with the LBS server as much as possible in order to limit exposure to location-sensitive information. Domingo-ferrer et al. [36] proposed a cooperative method for disturbing users’ location information by adding Gaussian noise. This method requests disturbed location information from other users and then forms a cloaking region according to that information. Rather than using the true location, the anonymous group’s density center, formed by cooperative users, is used as the anchor point to replace it and launch query requests. Shokri et al. [37] proposed an effective collaborative location privacy protection approach. Zhang et al. [38] proposed a cache and spatial K-anonymity-based privacy enhancement technique.

This strategy employs a multi-level caching method to reduce the possibility of user location information being disclosed. Niu et al. [39] created a privacy protection algorithm using dummy locations and cache awareness. The research on privacy protection techniques based on caching and collaboration focuses on three main areas: reducing cache overhead, improving the cache hit ratio and location privacy, and quantifying the QoS level. Another consideration is how to reduce the expensive communication cost caused by such a collaborative technique architecture.

#### 2.3.4. Location Privacy Protection Techniques Based on Anonymity

Methods based on anonymity to protect location privacy, such as k-anonymity and mix-zone, protect privacy by breaking the link between user identity and location data. The k-anonymity [40] technique ensures that the user’s location information cannot be differentiated from that of other k−1 users through generalization. As a result, attackers have a 1/k chance of discovering users’ true location. Stajano et al. [41] proposed the Mix-zone, which differs from the k-anonymity scheme. Attackers are unable to precisely pinpoint the user’s real location by frequently changing the user’s name or pseudonym in the anonymity area. In a variety of settings, anonymous approaches have been thoroughly researched and tested. However, this strategy raises concerns because maintaining the same level of anonymity in different scenarios is difficult.

**Figure 2:**
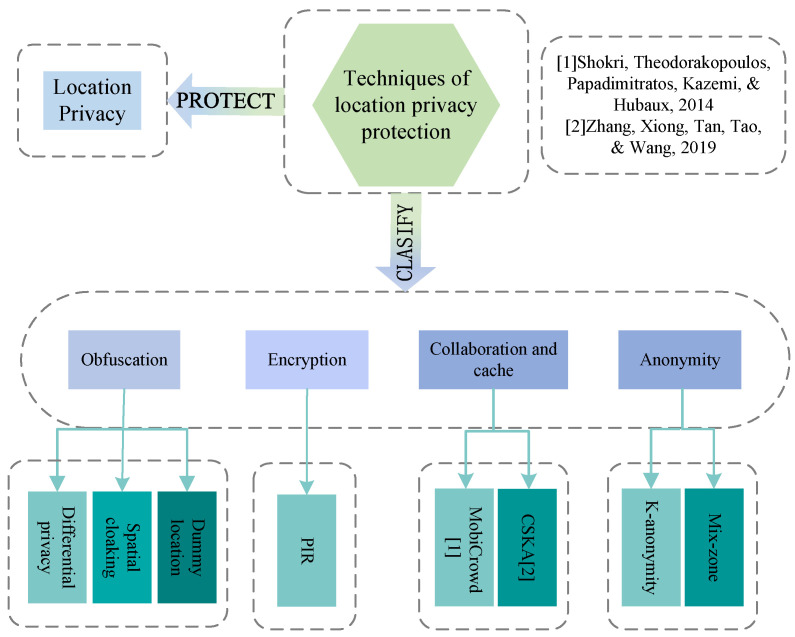
The relationship among location privacy, location privacy protection techniques, the obfuscation, and dummy location.

The relationship between location privacy, location privacy protection techniques, obfuscation, and dummy generation is depicted in Figure 2. Table 1 compares existing location privacy protection techniques in terms of LoP, outlining their main advantages and disadvantages. The system overhead of the four location-based privacy protection techniques is compared in Table 2. Given that different privacy protection techniques provide different benefits, we must adopt location privacy protection methods that are appropriate for the given application in order to protect the user’s location privacy.

Dummy is an important obfuscation method that has stimulated the interest of researchers both at home and abroad. This is becayse it is simple to implement, does not require a trusted third party, and can protect location privacy while maintaining accuracy. Furthermore, we can see that dummy has other advantages over other privacy protections in Table 1 and Table 2, such as low communication costs, low computation costs, and low storage costs.

## 3. Dummy-Based Attack Model

Malicious attackers aim to exploit various types of external information to find sensitive information about users, in addition to processing queries using various privacy protection mechanisms. However, the user’s location contains inherent “side information”, such as route information, human flow, and population distribution of the geographical region where the user is located [39,42]. Furthermore, attackers can obtain background knowledge in a variety of ways, including collaborative information systems, publicly available data aggregation, data brokers, data mining, and so on, in the age of Big Data and the Internet of Things.

Based on the attacker’s prior knowledge in two dimensions, namely temporal information and context information, attacks can be classified into context dimension attacks and temporal dimension attacks [43]. In the former case, the attacker only has a single snapshot of a user’s location, whereas in the latter case, the attacker has several locations collected over time or even a trajectory. We only consider the attack model on the context dimension in this paper because time is not taken into account. The most common threat to dummy-based location privacy protection techniques is background knowledge attacks in the context dimension. Such attacks can be classified into three types based on the attackers’ prior knowledge: location-distributed attack, probability-distributed attack, and semantic similarity attack. This section will summarize the attack model of dummy-based location privacy protection techniques.

### 3.1. Location-Distributed Attack

The location distribution attack is a type of attack method in which the attacker explores the location distribution characteristics in the user-specified cloaking area. It is classified into three types. One is that the location distribution of the cloaking area is overly concentrated, resulting in a small hidden area. For example, all of the locations are in the same neighborhood. However, although it successfully blurs users’ real locations, users’ location privacy cannot be adequately protected. Regarding the second type, the user’s true location is in the middle of the entire cloaking region, and the attacker can significantly reduce the user’s range [44]. For instance, all of the dummy positions are centered on the real location. In the third type, the real cloaking area shrinks as a result of the uneven location distribution caused by the attacker’s exclusion of some locations, which fails to meet the theoretical cloaking requirements. For example, if the majority of locations are distributed in a concentrated manner while one or two or a small portion of them are distributed in a relatively scattered manner, attackers can easily filter out those locations, reducing the original privacy protection intensity [45].

### 3.2. Probability-Distributed Attack

The probability distributed attack is defined as the attacker calculating historical query probability information by collecting historical service request records for all locations within a specific geographical region and over a specific time period [46]. When the probability distribution in the anonymous set generated by the user’s query request is uneven, the attacker filters out the dummy locations with a large gap, resulting in a failure to achieve the true location privacy protection effect. If the chosen dummy locations set includes several dummy locations in the middle of the lake with zero query probability, the attacker can simply deduce that they are dummy locations and filter them out.

### 3.3. Semantic Similarity Attack

The semantic similarity attack refers to the attacker’s speculation on the privacy information of users by parsing semantic information of locations in cloaking regions, such as behavior habits, health status, and professional attributes [47]. As long as all dummies’ query probabilities and the real location of the user’s query probability are equal or close, attackers can easily infer user behavior if all dummies in cloaking areas belong to the same kind of semantics.

## 4. Dummy-Based Location Privacy Protection Techniques

In this section, we outline the two system architectures of dummy-based location privacy protection techniques, then review the dummy-based location privacy protection techniques, and finally analyze and summarize how the dummy-based location privacy protection techniques choose dummies to handle background knowledge attacks.

### 4.1. System Architectures of Dummy-Based Location Privacy Protection

Dummy generation system architectures can be divided into two types: architecture with a third party and architecture without a third party, depending on whether a third party is deployed or not [48].

#### 4.1.1. Architecture with a Third Party

**Figure 3:**
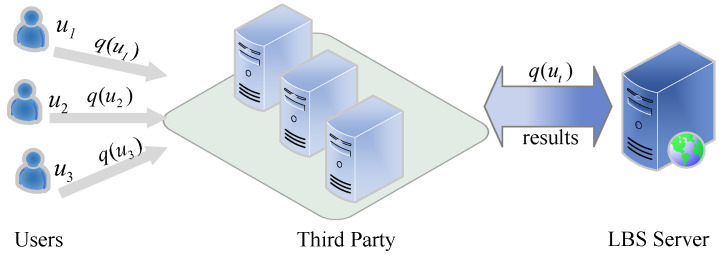
The architecture with a third party.

This architecture consists of users, a third party, and an LBS server. One or more servers represent a third party [49,50], and these are the servers that generate the dummy location set for the query user in order to mask the true location. Figure 3 depicts a third-party architecture. The primary responsibility of the third party is to collect and process user query requests, protect sensitive location information using privacy protection techniques, and then forward the processed query requests to the LBS server. After receiving these requests from the third party, the LBS server retrieves the database and transmits the matching result sets to the third-party servers. Finally, the requesting users receive the result sets from the third-party servers. Third-party servers, for example, create a cloaking zone with multiple users, and all users in the zone submit the same query to LBS. In this case, the LBS server is unable to determine who initiated the query and, as a result, is unable to find out which location is the original requesting location.

Obtaining a completely trustworthy third party, on the other hand, is difficult, and the “honest but curious” third party is vulnerable to a single point of attack and other vulnerabilities. As a result, the researchers have proposed an architecture that does not rely on a third party.

#### 4.1.2. Architecture without a Third Party

**Figure 4:**
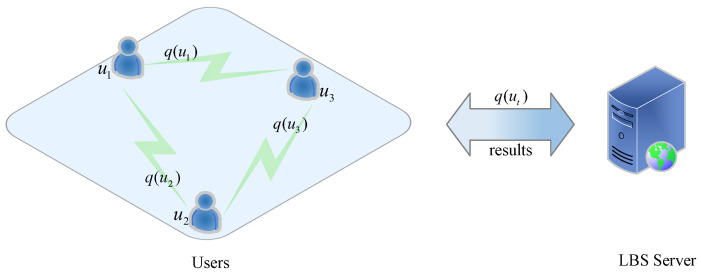
The architecture without a third party.

Figure 4 depicts the architecture in the absence of a third party, which consists of users and an LBS server.

The architecture requires that mobile devices carried by users have certain computational and storage capabilities that can be used to select dummy locations, create cloaking areas, and save map data within a certain range. The non-third-party architecture can be divided into two types based on whether or not users collaborate. In the first type, users’ location information is concealed in accordance with their privacy requirements [51]. For example, the Apple differential privacy team uses local differential privacy [52]. Users’ personal data can be randomized on their devices before being uploaded to the server, which can improve the user experience without infringing on privacy. In the second type, users collaborate for the sake of secrecy [53]. Tor, for example, is a volunteer-run distributed relay network that enables users to conceal their location while providing a variety of services. When using this method of obscuring through user collaboration, it is important to consider the additional communication cost between users as well as the risk of collusion attack [54].

### 4.2. The Dummy-Based Location Privacy Protection Techniques

**Figure 5:**
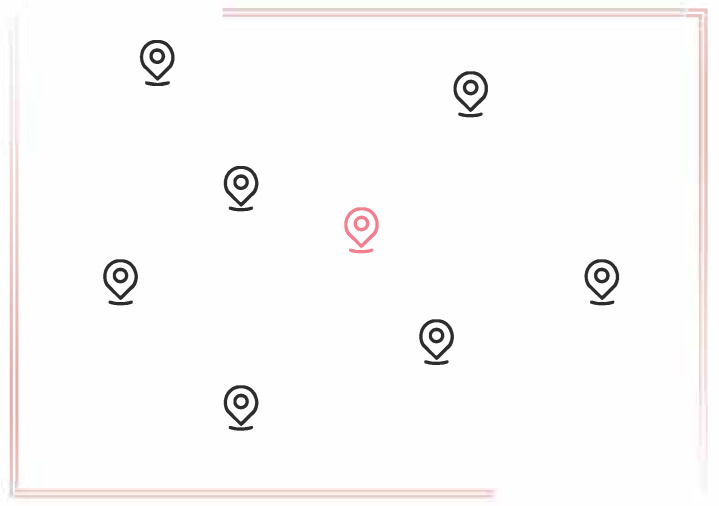
A cloaking area with k=8 users.

The dummy-based location privacy protection techniques select many dummy locations (assuming k−1 dummies) and send the same query request to the LBS server with the real location, making it difficult for the LBS server to distinguish the real ones from those *k* locations. However, if those dummies are chosen at random or without taking into account the attacker’s background knowledge, some of the dummy locations will be too large for the attacker to filter out, and the theoretical LoP will be impossible to achieve. Figure 5 shows a cloaking zone with k=8 users. The colorful one represents the user’s true location, whereas the black ones represent the user’s chosen dummy locations. The *k* locations cover the cloaking area.

In general, the higher the *k* value, the greater the privacy protection; otherwise, the lower the privacy security. When the value of *k* increases, the corresponding QoS decreases and the system overhead increases.

### 4.3. Algorithms of Dummy Location Selection

Researchers proposed a variety of approaches in the dummy-based locations’ selection to withstand the background knowledge attack, such as [55]. The main work of these studies is to choose appropriate dummy locations to construct a candidate set that protects users’ privacy effectively. The aim of dummy-based location privacy protection is to camouflage the user’s real location in the dummy locations concentration; thus, the quality of these selected candidate dummies is crucial to attaining the desired level of location privacy in the overall system. As a result, it is critical to reduce the distinguishability of real and dummy locations in all aspects; that is, we must choose dummy locations that can satisfy user desires while also protecting user privacy. In this subsection, we summarize and discuss the rules on dummy selection for dummy-based location privacy protection techniques.

#### 4.3.1. Take the Historical Query Probability of Locations into Consideration

The popularity of a location within a geographic location area over time is reflected by its historical query probability. The ratio of the number of times a location is queried to the total number of times all locations are requested in the global geographical area is used to calculate the historical query probability of a location in a certain period of time. For example, the following is the calculation formula for the historical query probability of location *i* inside a specific geographical area over time:

(1) $$q_{i}=\frac{times\;of\;queries\;in\;location\;i}{times\;of\;queries\;in\;all\;locations},$$

Because the LBS server has background information such as historical query probability of map locations, the server filters out dummy locations with obvious differences based on the probability distribution information of the candidate set, and thus the expected level of privacy protection cannot be achieved.

**Figure 6:**
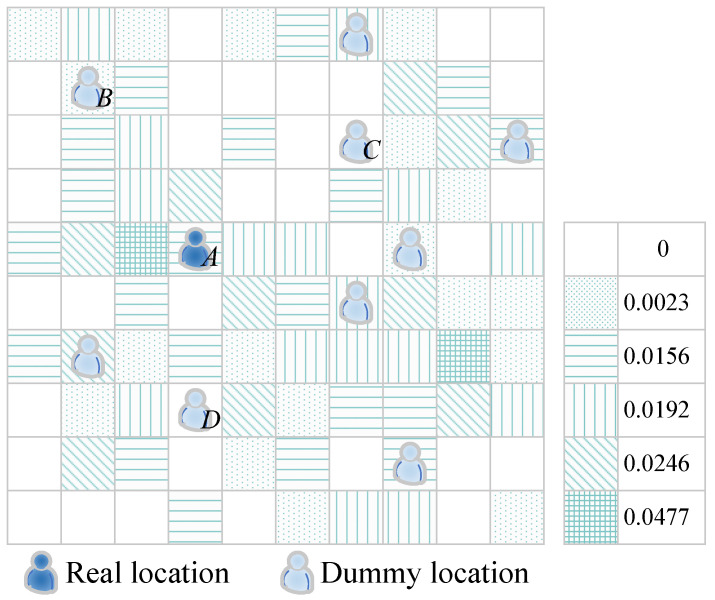
The historical query probability distribution of all locations.

If the server filters out *m* dummy locations, the likelihood of identifying the user’s dummy location increases from $\frac{1}{k}$ to $\frac{1}{k-m}$. In the entire map space, Figure 6 depicts the distribution of all locations and their historical query probability. Each little grid cell in the diagram represents a location. Varied shadow shapes portray different historical query probabilities, and the sum of the probabilities of all locations initiating query requests in the entire grid space is 1. Location *A* represents the user’s real location, whereas *B*, *C*, and *D* are the dummy locations that have been chosen. Because their historical query probability is smaller than the real location’s or even zero, the server can easily filter these dummy locations out.

Hara et al. [7] developed a dummy location selection algorithm that considers real-world environmental constraints and avoids dummy locations in inaccessible locations, such as the middle of a lake. However, this method only eliminates a small number of impossible locations, those where $q_{i}=0$. As a result, the dummy quality is poor, as is the LoP. In order to improve the quality of dummies and the LoP, the DLS algorithm chooses dummy locations that have the same probability as the real ones. It not only keeps these q=0 locations at bay, but it also reduces the difference in query probability between the real ones and dummies. In the literature [56], the greedy algorithm idea is used to select dummy locations so that the new location set composed of each new dummy location and the previously selected dummy locations have the best hiding effect. Other authors [57,58] have employed an information entropy-based method, with the historical query probability as a variable, to choose dummy locations. In [57,58], the set of dummy locations with the highest entropy value acts as the final set of candidate dummy locations. Because the historical query probability of each location over time is insufficient to convey the prevalence of each location, [59] introduced the concept of “current query probability”, which was used to replace historical query probability as the criterion for selecting dummy locations. Users choose different geographical regions for different time periods, with each location’s current query probability being different. As a result, the “historical query probability” is more diverse, posing a greater challenge to attackers.

#### 4.3.2. Taking the Physical Dispersion of Locations into Consideration

The physical dispersion between locations describes the spatial distribution of locations. The obscuring of users’ true locations will also perform poorly if an attacker learns this background knowledge in order to carry out location distribution attacks on them. As a result, selecting dummy locations solely on historical query probability is insufficient. In practical applications, physical dispersion between locations should be highlighted.

**Figure 7:**
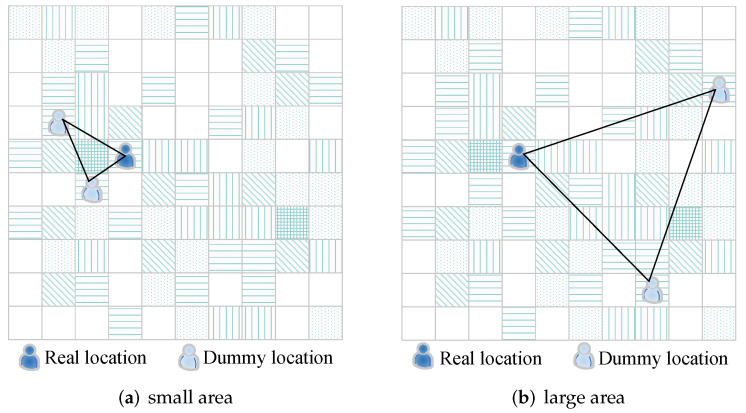
The physical dispersion situation between dummies and the real location.

If the physical dispersion between locations is too small, the cloaking area will be too small. The cloaking area, as shown in Figure 7a, is small, allowing the attacker to quickly deduce that the real user is in a very small area. As a result, something like Figure 7b would be preferable because it provides a larger cloaking area for the real user. Simultaneously, the query probability of those chosen dummy locations is not too far off from the user’s actual location. As a result, when selecting dummy locations, the spatial distribution of the k−1 dummy locations and the real ones should be guaranteed, while the historical query probability should be the same or similar.

To meet the requirements of physical dispersion of locations, Niu B. et al. [8] proposed a method for selecting candidate locations based on virtual circles and virtual grids. Because the user’s true location is likely to be close to the center of the local map, the virtual circle algorithm may have performed poorly in terms of privacy. To provide a more obscured area, a virtual grid-based algorithm was introduced, which ensures that candidate locations are distributed fairly evenly around the true location and that the size of the cloaking area meets user needs.

**Figure 8:**
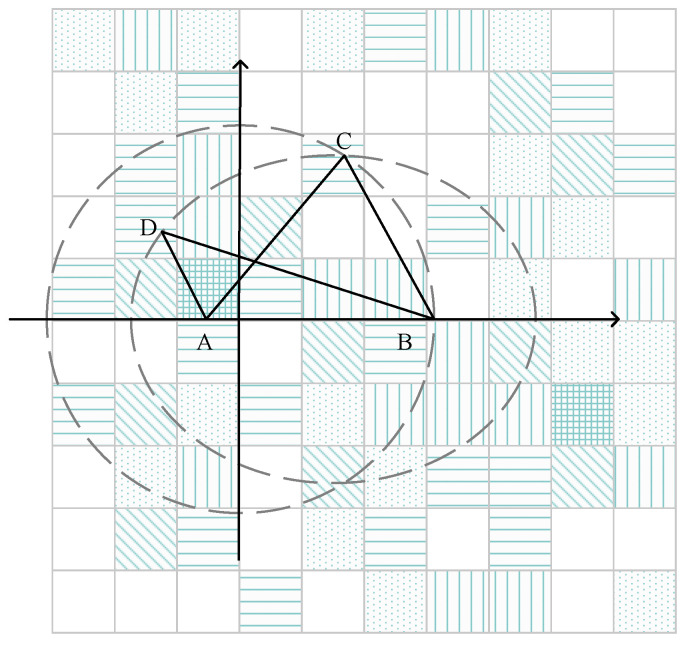
The enhanced DLS.

In order to achieve physical dispersion between locations, Niu B et al. proposed the enhanced DLS algorithm in reference [42]. They argued that the product of locational distances was more accurate in depicting locational dispersion than the sum of locational distances. As Figure 8 shows, CA+CB=DA+DB while CA·CB>DA·DB; as a result, we would prefer to choose *C* over *D* from the perspective of privacy. They used a multi-objective optimization model as well, where the probability and physical dispersion of locations are considered simultaneously to pick the best candidate set of dummy locations.

In addition to the previous research on location dispersion in physical space, there are numerous studies on how to portray the physical distance, such as the effective distance [60] or the road network distance [61,62], between two locations. The idea of effective distance was developed by Xu et al. [60] to characterize the distribution features of locations, and the effective distance between these two locations was defined as the shortest distance between the current location and any other location. Consider real user $u_{r}$ and any other user $u_{i}$; their coordinates are $\left(x_{r},y_{r}\right)$ and $\left(x_{i},y_{i}\right)$, resulting in an effective distance of

(2) $$d\left(u_{r},u_{i}\right)=\min|u_{r},u_{i}|=\min\sqrt{{\left(x_{r}-x_{i}\right)}^{2}+{\left(y_{r}-y_{i}\right)}^{2}}$$

**Figure 9:**
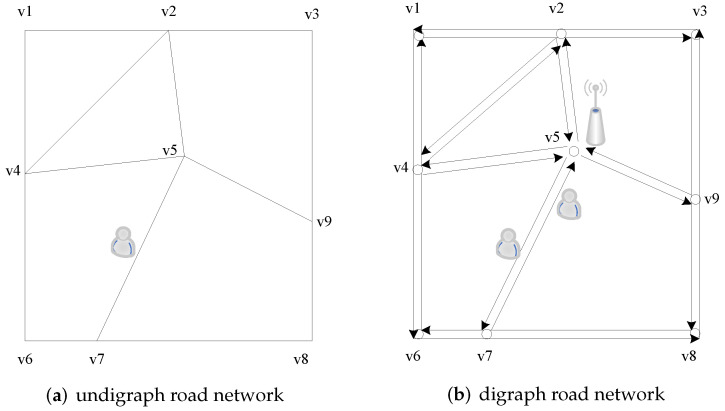
Undigraph/digraph road network.

It is apparent from the effective distance calculation formula that the essence of the effective distance specified by them is the Euclidean distance. Despite the fact that the article is based on a real-world road network, Euclidean distance is nevertheless employed to measure location distribution features. Chen et al. [62] proposed a privacy protection method for the road network in response to the fact that the distance between any two points in real life is not a simple linear distance (Euclidean distance), and that users’ activities are more restricted to the planned road. This approach requires that the number of road sections in the selected dummy sites satisfy the value given by the user in order to attain the purpose of physical dispersion between the selected dummy locations when picking dummy locations. This road network, however, is an undigraph road network model, which is insufficiently realistic for real-world road network simulation, as illustrated in Figure 9a. Zhou Changli et al. proposed a location privacy protection approach based on the digraph road network architecture (as shown in Figure 9b), in which an anchor point (dummy location) was used to replace users’ real locations when initiating query requests. However, when choosing the anchor point, the historical query probability of the anchor and its geographical spatial distribution link with a real user were not taken into account.

#### 4.3.3. Taking the Semantic Diversity of Locations into Consideration

A location’s semantic information refers to its semantic properties, such as hospitals, restaurants, banks, schools, parks, and so on. Semantic features can be extracted using context information. The greater the number of location semantic features collected, the more accurate the semantic categorization, and the greater the ability to protect users’ location privacy. Consider the semantics of a user’s location for “hospital” which implies semantic information on the user’s health, professional property, and so on. Because the user’s state of health or professional attributes belong to the users of the important content of privacy, semantic information must be considered when selecting dummy locations.

Bostanipour [63] presented a method for combining obfuscation location information with semantic information to ensure that many semantically identical locations are cloaked, therefore preventing attackers from performing semantic inference attacks. The locations derived using this method, on the other hand, are semantically related to those of real users. For example, the real user’s semantic tag is “Pizza Place”, but the cloaking region includes venues such as “Noodle House” and “Hamburger Palace”, all of which belong to the parent semantic tag “Restaurant”. As a result, such a method is still vulnerable to semantic inference attacks.

In order to achieve semantic diversity, each location in the candidate dummy set should have a diverse set of semantic properties as much as possible. While representing semantic differences between locations is a challenge, Zeng et al. proposed the similarity of two semantic location types using Euclidian distance to calculate [64]. Tian et al. measure semantic distance based on the intersection and union of a location’s semantic attributes:

(3) $$sem_{dist}\left(A,B\right)=\frac{\left[sem_{A}\cup sem_{B}\right]-\left[sem_{A}\cap sem_{B}\right]}{sem_{A}\cup sem_{B}}$$

**Figure 10:**
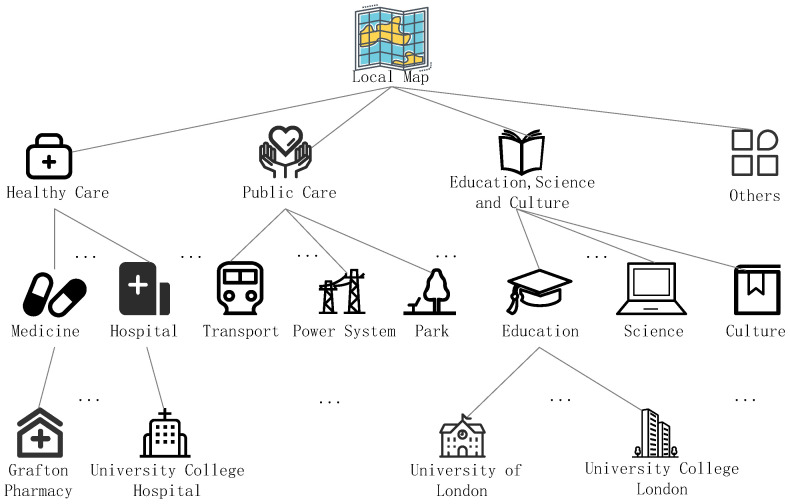
The location semantic tree.

This then transforms the results to show semantic similarity [65]. Using Euclidean distance and the relationship between sets to quantify semantic difference not only consumes a lot of effort but also weakens the algorithm’s efficiency. Another author [9] created a location semantic tree (LST) to arrange all locations, as shown in Figure 10, in order to achieve semantic similarity control that can serve the tailored needs of users and increase the efficiency of algorithm execution. The most fundamental semantic information is stored in the leaf nodes of the location semantic tree, and the hop number between the leaf nodes is used to calculate the semantic distance, which is then used to calculate the semantic difference degree. This approach can rapidly find and categorize the semantic associations of all locations in a specified geographical area.

When there are many different semantic varieties in a given geographical area and there are crossover circumstances, the depth and breadth of the semantic tree grow quite large, decreasing search efficiency. As a result, the location semantic tree is not ideal for such scenarios.

### 4.4. Summary

In general, we classify the existing dummy location selection methods into three categories according to the types of attacks they can defend against, as shown in Table 3. The first category of selection method can successfully defend against the probability similarity attack. The second category of the selection method can effectively prevent physical distribution attacks launched by attackers on the distribution pattern of locations. The third category of selection method can make it difficult for attackers trying to obtain cracking clues from the semantic information of locations. Different methods have different characteristics, and we can select relatively appropriate methods according to our own needs and purposes when using these methods to select dummies to construct dummy location’s set.

An overall comparison of random selection and other selection schemes that consider different factors when selecting dummies is shown in Table 4. In Table 4, we observe that different schemes can choose different system structures and take different factors into account to design different schemes according to their own purposes and needs. As a consequence, the types of attacks they can defend against are not the same, and, of course, the corresponding computational overheads are somewhat different. Dummy location selection methods that take into account query probability, physical dispersion, and semantic diversity yield better security than random selection with a relatively small computational overhead. Furthermore, depending on different selection factors, the attacks that can be defended against are varied when selecting a dummy location. When a dummy location is chosen, the more factors are taken into account, the better the privacy protection effects of the scheme are strengthened, while the difference in computing overhead is not readily apparent. As a result, schemes increasingly seek to take more factors into account when selecting dummies. They are no longer always based on a single factor, such as [21,66], which incorporates two factors, and three factors are considered simultaneously in the literature [46]. As the research goes further, new factors are discovered and considered, and new rules are established in [67,68].

## 5. Conclusions

In this article, we provide a review of dummy-based location privacy protection techniques for LBS. First, we distinguished the relationship between the LoP, QoS, and system overhead. At the same time, we made an overall comparison of several representative methods of location privacy protection techniques. We described the merits of dummy-based location privacy protection on LBS. Meanwhile, a summary of the major attacks on dummy-based location privacy protection techniques was also included.

Second, we systematically and comprehensively analyzed and summarizde the ways of selecting dummies on three aspects, namely the query probability, the physical dispersion, and the semantic diversity of locations.

Third, we provided an overview of the methods for achieving query probability, physical dispersion, and semantic diversity while choosing dummies. Furthermore, the different privacy protection advantages of different selection rules when choosing dummies can be seen from a comparative analysis. The results of this comparative analysis can benefit both users and researchers who are studying this field. When the requesting service needs to construct a hiding area that hides their true location, the user can refer to this comparative evaluation to choose a dummy-based location privacy protection method that better meets their needs. Moreover, researchers studying this area can gain a better understanding of dummy-based privacy protection schemes from the results of this comparative analysis. They can also get to know the challenges posed by the expanding background knowledge of attackers and the intersection between LBS and other emerging technologies.

Dummy location selection approaches that took into account new circumstances in the selection of dummies emerged as research progressed. There are still some significant issues to be resolved and perfected in the area of dummy location selection.

First, as new technologies such as social networks, edge computing, and federal learning have been advanced, new privacy concerns have also emerged.

- Because location acquisition technology is becoming more widely available, it is now possible to add geo-information to already-existing social networks, which has facilitated in the emergence and expansion of LBSN. LBSN, a combination of LBS and social networks, involves a range of personal private information, such as shared common locations, personal interests, daily behaviors and activities, etc. [69].
- LBS@E [70] delocalizes LBSs and retrieves local information from nearby edge servers around them instead of the cloud. Consequently, it tackles the location privacy problem innovatively. However, LBS@E brings new challenges to location privacy. Mobile users can still be localized to specific privacy areas jointly covered by edge servers accessed by mobile users. The small privacy area puts the mobile user’s location at risk of similarity.
- Ref. [71] uses federated learning to select the best location privacy protection mechanism (LPPM) for each user according to the real location and the user’s configuration, which avoids the direct use of the real location information. Nevertheless, it is vulnerable to poisoning attacks and untrusted users who intend to add a backdoor to the model [72] or defend against attacks on model information leakage [73].

Second, existing dummy-based solutions do not account for all aspects of real-world privacy protection [74], and there is a significant gap between theoretical and real-world privacy protection effects. According to Sun et al. [75], attackers can also rule out impossible dummy locations by determining whether users can reach the query location in a reasonable amount of time from their current location.

Third, dummy-based approaches that focus on the spatio-temporal correlation of location are commonly used in trajectory privacy protection, which poses new challenges in trajectory privacy. Zhao [76] assumes that all users(dummies) involved are trustworthy and report their real locations. However, it is often not the case in reality. There are untrusted users who conduct location injection attacks (LIAs) in continuous LBS queries. Zhen [77] found that the trajectory data were published without proper processing. A great amount of work has been devoted to merging one’s own trajectories with those of others, without protecting the semantic information about the location. In continuous LBS queries, users can obfuscate their true query location by selecting dummy locations and predicted locations, thus improving their privacy. However, selecting a large number of dummies for each query can increase the query cost of the system and influence the accuracy of the predicted location [78].

## Figures and Tables

**Table 1:** The comparison among four privacy protection techniques.

LPPT ^1^	RM ^2^	LoP ^3^	TTP
Obfuscation	Dummy		
	Spatial Cloaking	low	yes
	Differential Privacy		
Encryption	PIR	high	no
Collaboration and Cache		medium	no
Anonymity	K-anonymity	medium	yes
	Mix-zone		

^1^ LPPT: location privacy protection techniques. ^2^ RM: representative method. ^3^ LoP: the level of protection privacy.

**Table 2:** The cost of four privacy protection techniques.

LPPT	Precision Loss	Communication Cost	Computation Cost	Storage Cost
Obfuscation	high	low	low	low
Encryption	low	low	high	medium
Collaboration and Cache	medium	high	low	high
Anonymity	medium	medium	high	medium

**Table 3:** Selection methods of dummy on query probability similarity, physical dispersion and semantic diversity.

Category	Reference	Methods of Selection
Query probability similarity	[7]	avoids dummies with $q_{i}=0$
	[42]	dummies have the same probability as the real ones
	[58]	information entropy-based
	[59]	current query probability
Physical dispersion	[8]	virtual circles and virtual grids
	[42]	the product of locational distances
	[60]	the effective distance
	[62]	the road network distance
Semantic diversity	[9]	location semantic tree
	[64]	Euclidian distance
	[65]	the intersection and union of a location’s semantic attributes

**Table 4:** Summary of dummy selection.

Selection Method	Reference	CO ^a^	Architecture		Attack		
			TTP	Non-TTP	AoQ ^b^	AoD ^c^	AoS ^d^
Random Selection	[6]	O(klogk)		*√*			
Considering Q	[42]	O(k)		*√*	*√*		
Considering D	[7]	Null		*√*		*√*	
Considering S	[62]	Null	*√*				*√*
	[64]	Null	*√*				*√*
Considering Q+D	[8]	O(k)		*√*	*√*	*√*	
	[9]	O(logk)		*√*	*√*	*√*	
	[58]	$O(\alpha \log_{2}\alpha )$		*√*	*√*	*√*	
	[60]	$O(k^{2}+IJU)$		*√*	*√*	*√*	
Considering D+S	[59]	O(k)		*√*		*√*	*√*
Considering Q+S	[65]	O(It·k)		*√*	*√*		*√*
All of them	[18]	O(logN)	*√*		*√*	*√*	*√*

^a^ CO: the computation overhead. ^b^ AoQ: the attack of query probability; Q: Query probability. ^c^ AoD: the attack of location distribution; D: Location distribution. ^d^ AoS: the attack of semantic similarity; S: Semantic similarity. Notes: *k*: the number of dummies; *α*: (*ω* + *m*) log(*ω* + *m*), *ω* = (*maxtier* − 1)(1 − *e*)*m*, *m*: the number of dummies candidate set, *maxtier*: the max times of iteration; *IJ*: an area is divided into *I* × *J* cells; *U*: the number of services; *It*: the times of iteration; *N*: the total number of users in the region to be clocked.

## Abbreviations

The following abbreviations are used in this manuscript:

LTTP	location privacy protection techniques
LoP	the level of privacy protection
RM	representative method
QoS	the quality of service
CO	computation overhead
AoQ	attack of query probability
Q	query probability
AoD	attack of location distribution
D	location distribution
AoS	attack of semantic similarity
S	semantic similarity
